# Spatial Insights in Cardiovascular Aging

**DOI:** 10.14336/AD.2025.0272

**Published:** 2026-04-28

**Authors:** Zhongling Dai, Huiqin Ding, Quan Zhang, Liyao Fu, Shi Tai

**Affiliations:** ^1^Department of Cardiology, The Second Xiangya Hospital of Central South University, Changsha 410011, China.; ^2^Department of Blood Transfusion, The Second Xiangya Hospital of Central South University, Changsha, China.

**Keywords:** spatial omics, cardiovascular aging, spatial heterogeneity

## Abstract

Spatial omics provides unprecedented insights into how the cardiovascular system is spatially organized and how cellular phenotypes are distributed. Researchers have been able to clarify the complex spatial architecture of the cardiovascular system and how cellular phenotypes are distributed during the aging process by integrating data from spatial omics. In addition, this new technology has revealed previously hidden patterns of gene expression and cellular communication that were not detected using traditional bulk omics approaches. In this review, we explore the contribution of spatial omics in clarifying the molecular mechanisms that influence cardiovascular aging, highlighting the importance and application of spatial omics in unraveling the spatial heterogeneity within the aging cardiovascular system. This will help us understand the molecular mechanisms implicated in age-related cardiovascular diseases.

## 1. Introduction

**Figure 1.**
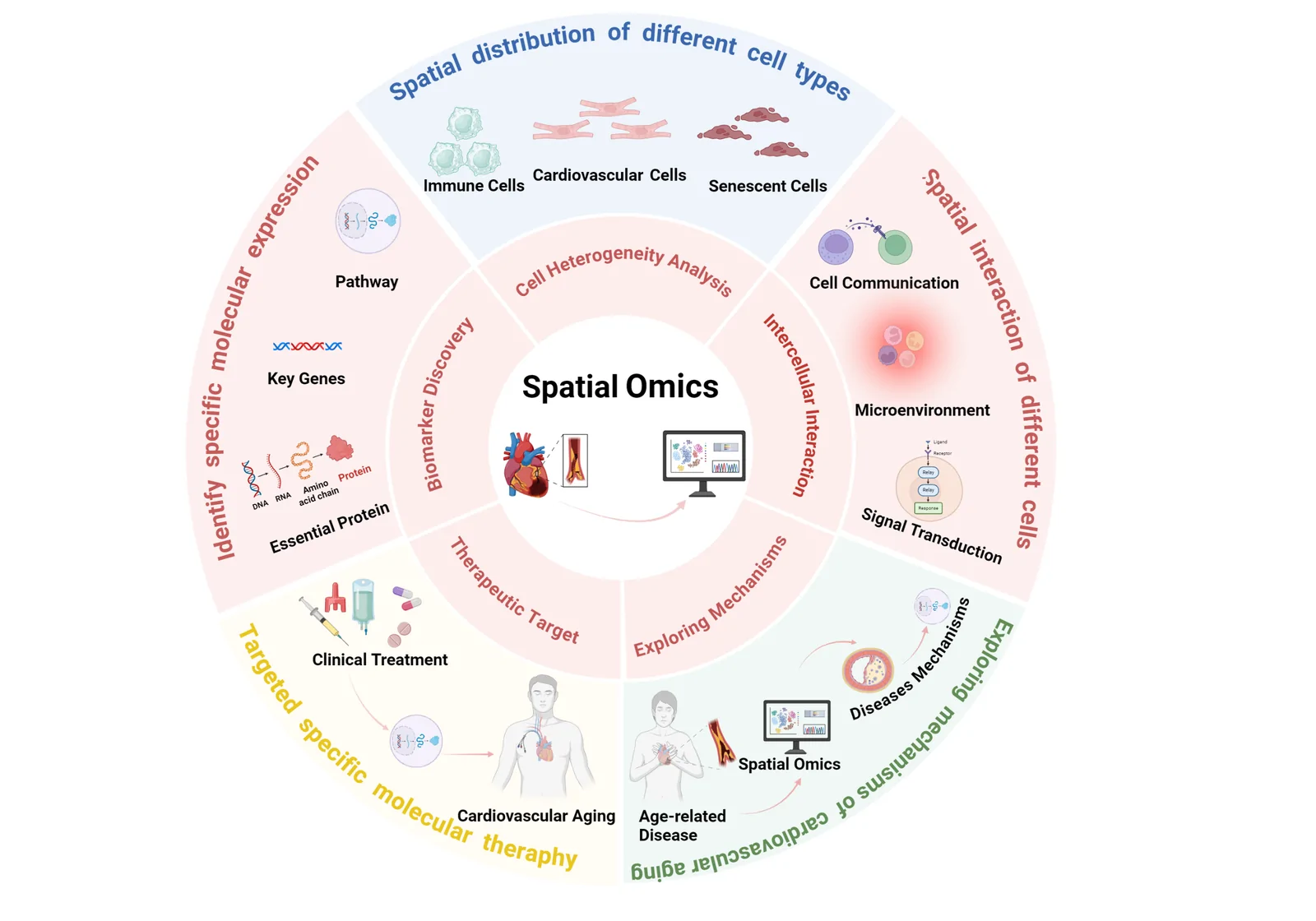
**Spatial omics Functions**. Spatial omics technologies, by preserving the spatial context of cells, facilitate a comprehensive understanding of the distribution of tissue and cellular phenotypes in cardiovascular aging. They also elucidate the mechanisms of intercellular communication and identify novel biomarkers, thereby providing innovative therapeutic approaches for the treatment of aging-related cardiovascular conditions.

Cardiovascular diseases remain a leading cause of morbidity and mortality worldwide， intricately linked to many risk factors. Among these, age emerges as the most pronounced and irreversible determinant of cardiovascular health [1-4]. As the age of the global population increases, the prevalence of age-related cardiovascular conditions rises, underscoring the imperative to elucidate the intricate biological processes underlying cardiovascular aging [4,5]. Recent studies have explored eight molecular markers of cardiovascular aging and potential therapeutic intervention targets, which reveal that the complex interplay between cellular senescence, telomere shortening, oxidative stress, and altered metabolic pathways progressively deteriorates cardiovascular function with advancing age [6-10]. Cardiovascular aging involves various changes at the molecular, cellular, and tissue levels. However, our current understanding of these processes is incomplete because traditional omics approaches are limited and often fail to capture the spatial context of molecular changes. The emergence of spatial omics has partially addressed this limitation. Unlike conventional omics technologies, spatial omics is capable of measuring a diverse array of molecular characteristics of cells, including genes, transcripts, proteins, and metabolites, within their native tissue microenvironments [11]. Importantly, spatial omics not only focuses on the types and quantities of molecules but also reveals their precise locations and spatial distributions within cells and tissues. Recently, spatial omics technology has become a crucial tool in studying spatial changes in various conditions, such as tumors, kidney failure, and cardiovascular diseases [12-15]. Hence, we posit that the advancement of spatial omics technologies holds the potential to not only advance our investigations into the spatial backgrounds of these molecular changes underlying cardiovascular aging but also facilitate the identification of novel biomarkers and therapeutic targets. Integrating spatial omics into cardiovascular aging research significantly enhances our comprehension of this intricate process. This technology enables the observation of cellular interactions within the microenvironment and elucidates how these interactions are modulated by cellular spatial positioning [16,17]. Moreover, by integrating single-cell transcriptomics with spatial information, spatial omics provides unprecedented clarity for visualizing gene expression patterns within the complex tissue architecture [11,18]. In summary, spatial omics, through the construction of molecular maps of cardiovascular tissues, unravels the molecular mechanisms of cellular senescence and tissue remodeling (Fig. 1). It has the potential to revolutionize our understanding of the aging cardiovascular system and to elucidate how specific cell populations influence cardiovascular function through inherent spatial heterogeneity.

This review aims to underscore the significance of spatial omics in elucidating the cardiovascular aging mechanisms, evaluate the technological advancements that facilitate its application, analyze the findings derived from spatial omics studies, and explore future directions for research and therapeutic interventions. The scientific community is poised to integrate spatial omics data with other multi-omics datasets to gain deeper insights into vascular aging. This will help create innovative diagnostic tools and targeted therapies to address cardiovascular challenges in aging populations.

## 2. The Biology of Cardiovascular Aging

**Figure 2.**
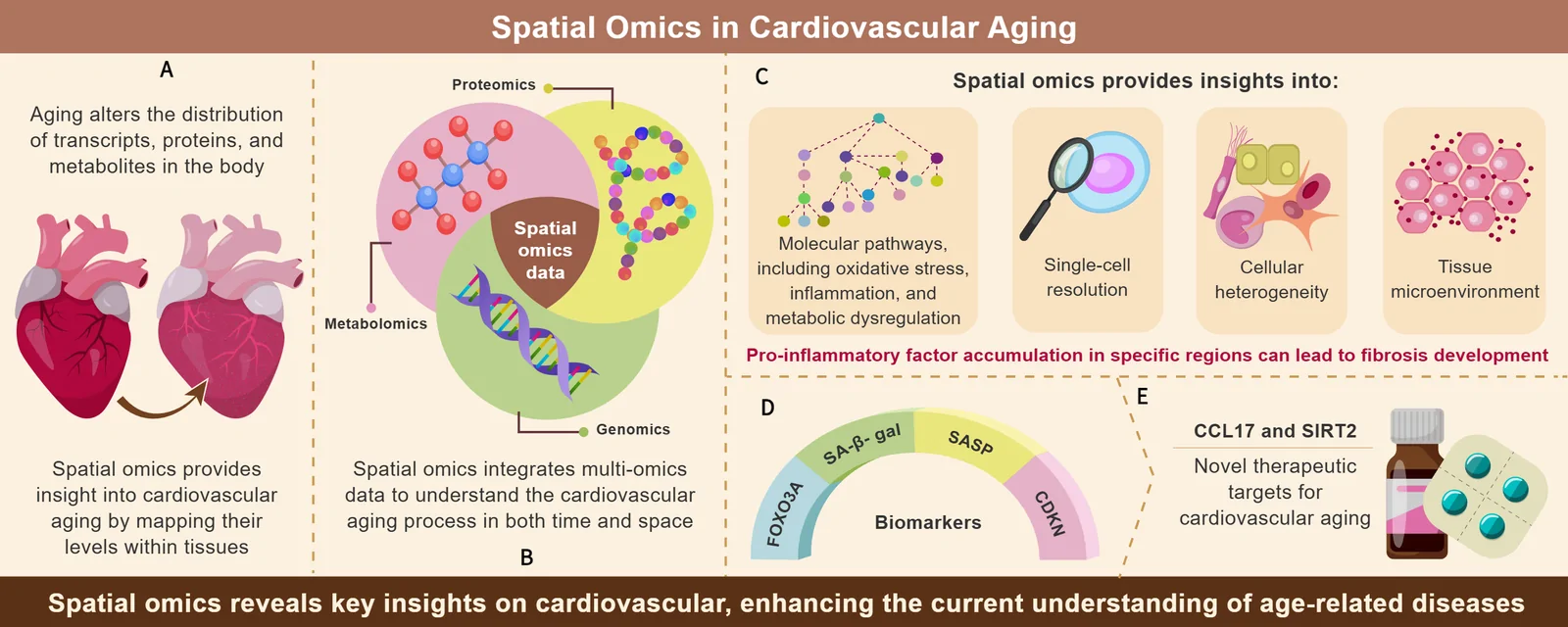
**Spatial omics in Cardiovascular aging. (A)** Aging alters the distribution of transcripts, proteins, and metabolites in the body.**(B)** Spatial omics integrates multi-omics data to understand the cardiovascular aging process in both time and space.**(C)** Spatial omics provides insights into molecular pathways, including oxidative stress, metabolic dysregulation, single-cell resolution, cellular heterogeneity, and tissue microenvironment.**(D)** Biomarkers include SASP, SA-β-gal, CDKN, and FOXO3A.**(E)** CCL17 and SIRT2 are novel therapeutic targets for cardiovascular aging.

Cardiovascular aging is characterized by the senescence of various cardiovascular cell types and tissues [9,19], thereby potentially increasing the susceptibility to cardiovascular diseases, such as atherosclerosis, hypertension, and heart failure [1,6]. Presently, the identification of biomarkers for early detection and the development of targeted interventions, such as senolytics to remove senescent cells or geroprotectors to mitigate age-related changes, remain central to experimental therapies [20]. Several senescence-associated markers have been identified, including cyclin-dependent kinase inhibitor (CDKN), senescence-associated beta-galactosidase, and senescence-associated secretory phenotype (SASP) (Fig. 2D) [21]. Researchers from the SenNet Consortium in the United States have proposed, for the first time, the use of aging markers, artificial intelligence, and cyclic markers to identify and characterize senescent cells [21]. However, the identification, characterization, and isolation of senescent cells *in vivo* remain challenging, particularly due to the lack of specific markers for senescence phenotype, which makes it challenging to distinguish certain markers from other similar non-senescence-related markers. Additionally, various biological processes occurring simultaneously may complicate the establishment of causality if the experimental background of aging markers and inflammation or fibrosis overlap [21]. Therefore, using the spatial omics principle to accurately locate new specific aging markers and to distinguish other types of cellular changes that may occur during aging is particularly important. Furthermore, the principal investigators also highlighted that integrating morphological differences with advanced imaging platforms to identify senescent cells will significantly advance our understanding of cellular senescence in the near future [21].

Overall, aging is a multi-dimensional process involving genes, proteins, metabolites, and their interactions. Spatial omics, by combining multi-omics data, facilitates the identification of novel therapeutic targets and provides valuable insights into clinical strategies. These efforts are aimed at promoting cardiovascular health and extending lifespan (Fig. 2A). However, translating the emerging knowledge of molecular mechanisms into effective treatments and preventive measures for the aging population remains a challenge.

## 3. Spatial Omics - A Technological Revolution

### 3.1 Overview of spatial omics

Spatial omics have made significant advancements in the field of aging research. It allows for the investigation of spatiotemporal relationships between different tissues and organs during biological aging. Furthermore, it helps researchers in analyzing the spatial distributions of cells, molecules, or genetic information within biological tissues [22]. Such advancements uncover the molecular mechanisms of aging and improve our understanding of the aging spatiotemporal characteristics. Examples include aging of the liver, kidney, ovaries, and brain [23-27]. Additionally, spatial omics enable the examination of spatiotemporal relationships between different organs and reveal the spread and interactions of the aging process throughout the entire biological system. For example, senescent bone marrow-derived monocytes can propagate aging to multiple tissues via extracellular vesicles [28]. The integration of spatial resolution techniques with multi-omics data through spatial omics has considerably contributed to the understanding of the complex molecular dynamics in biological aging. This interdisciplinary field combines spatial information with abundant omics data at different time points, providing a comprehensive spatiotemporal perspective on individual cellular systems [15]. In this review, we summarize the basics of spatial omics and explore how these cutting-edge technologies provide ultra-high-resolution insights into the interactions between cells and their microenvironment.

### 3.2 Basic principles and methods of spatial omics

Spatial omics technologies are based on the principle of preserving the spatial context of cellular components within tissues and analyzing their molecular profiles. The principal methodologies include spatial transcriptomics (ST), genomics, metabolomics, and multiplexed antibody-based spatial proteomics (STP), which includes advanced techniques such as imaging mass cytometry (IMC), multiplexed ion beam imaging (MIBI), and CO-Detection by IndEXing (CODEX) [11]. Among these, ST and STP represent two major focal areas within this domain. ST employs array-based, or spatially encoded, microfluidic devices to capture and analyze the DNA or RNA of individual cells located at precise coordinates within tissue sections [29,30]. This approach facilitates the concurrent measurement of gene expression across thousands of cells, which maintains their spatial architecture [29,30]. Notably, its analytical framework predominantly relies on techniques such as micro-dissection, combinatorial fluorescence *in situ* hybridization (FISH), single-molecule imaging, *in situ* sequencing of mRNA, and spatial barcode methodologies. Among these, single-molecule FISH is the gold standard for RNA quantification because it is highly sensitive and detects transcripts present in exceedingly low amounts [11]. In addition, barcode-based ST employs spatial barcode DNA oligonucleotide arrays to systematically capture tissue RNA, thereby enabling unbiased whole-transcriptome sequencing of RNA-containing entities [11]. *In situ* sequencing directly visualizes mRNA molecules within their native tissue context by hybridizing fluorescently labeled probes to target RNA, which facilitates spatial mapping of gene expression [31,32]. Gene expression is characterized by spatiotemporal heterogeneity, which is regulated by precise mRNA metabolism and time-dependent transport mechanisms. This integration of high-throughput sequencing and spatial localization offers a powerful tool for investigating the spatiotemporal heterogeneity of gene expression and enables the comprehensive characterization of single-cell transcriptomes in a spatiotemporally coherent manner. Despite significant advancements in these technologies, current transcriptomics methods remain incapable of concurrently capturing the spatiotemporal dependencies of RNA signatures and capturing the dynamic flow of gene expression. Therefore, developing highly multiplexed, spatially precise, and temporally resolved mRNA sequencing methods remains a formidable challenge [33].

The STP technology combines mass spectrometry with imaging techniques to explore the spatial distribution and functionality of proteins in cells or tissues [34]. It allows the subcellular localization of multiple proteins and reveals single-cell variability, dynamic protein translocations, and changing interaction networks [35]. Moreover, it facilitates the identification of protein expression changes associated with aging progression, thereby providing novel research targets. The core of STP technology encompasses antibody-based multiplexed imaging methods, such as IMC and MIBI. IMC integrates the high-dimensional analysis capacities of flow cytometry with the spatial resolution of imaging [11]. This technique uses metal isotope labeling to quantify multiple proteins simultaneously while retaining the tissue morphology [11]. MIBI serves as a high-resolution variant of IMC and employs secondary ion mass spectrometry to map the spatial distribution of elements and isotopes within tissues, thereby enhancing our understanding of cellular composition and functionality [11]. Additionally, the CODEX technology significantly enhances the capacity for multiplexed protein marker detection. It utilizes oligonucleotide barcodes specifically conjugated to each antibody, coupled with fluorescent dyes required for imaging. This approach allows for the iterative detection of multiple proteins within a single sample [11].

### 3.3 The connection between spatial omics and aging

The distribution of transcripts [36,37], proteins [38,39], metabolites [40,41], and epigenetic modifications undergo significant alterations as individuals age [42,43]. In response, researchers are increasingly utilizing single-cell optical methods to attain finer resolution and more comprehensive datasets [44]. These advanced techniques provide a high-throughput approach to understanding the complexities inherent to various cell types and individual differences [45,46]. However, these methods require tissue dissociation, which may obscure important cellular complexities, including spatial contexts and cell-cell interactions [46-48]. Consequently, integrating spatial omics technologies with single-cell omics data is essential for a holistic comprehension of aging (Fig. 2B) [48,49]. For example, Hahn et al. employed ST alongside single-cell sequencing to delineate the spatiotemporal transcriptome of the aging mouse brain [49,50], revealing the heterogeneous patterns of gene expression across spatial and temporal dimensions by sectioning tissues anatomically and analyzing tissues at distinct temporal intervals [50]. Similarly, Hao Y et al. investigated the role of DNA methylation patterns and epigenetic clocks in aging by analyzing epigenetic modifications in tissue slices at multiple time points [51]. Epigenetic clocks refer to DNA methylation patterns, which serve as biological markers of aging and indicators of biological age [52]. They can be utilized to predict biological age, assess aging rates, identify aging risk factors, assess anti-aging interventions, and predict the incidence of age-related diseases by analyzing specific age-related CpG sites [51-53]. Additionally, a novel spatial omics technique called Spatial-CUT&Tag allows for genome-wide analysis of histone modifications at a single-pixel resolution on frozen tissue sections without the need for tissue dissociation [54]. This facilitates the investigation of epigenetic mechanisms underlying tissue development at both spatial and genome-wide levels [54]. Furthermore, it has successfully detected various organ-specific cell types, including cardiomyocytes. It has been used in spatial and cell type-specific chromatin modifications analysis in mouse embryonic and postnatal brain development [54,55]. Aging is widely known to significantly alter the metabolic processes of various organs, leading to metabolic remodeling in age-related diseases such as cardiovascular diseases [56]. Furthermore, spatial metabolomics (STM) uncovers metabolic changes related to aging by precisely tracking the spatiotemporal dynamics of metabolites [49]. For instance, Walker et al. utilized STM technology to generate the spatiotemporal metabolite map of the mouse brain throughout the aging process, revealing noteworthy metabolic variations across diverse brain regions and developmental stages [57]. Notably, Jolanda et al. proposed integrating clinical phenotypes (including age and gender) with multi-omics and imaging data to uncover the specific metabolic remodeling in cardiovascular diseases and develop targeted therapies [58]. The aging process induces significant alterations in protein expression and modifications. STP enables precise spatiotemporal analysis of these changes [59,60]. It reveals the interaction networks of age-related proteins and facilitates the discovery of novel aging targets [49,60]. For instance, Hosp et al. employed STP to study the significant reconfiguration of the soluble brain proteome during Huntington's disease progression [61].

### 3.4 Advantages in cell-cell interactions and aging microenvironments

#### 3.4.1. Preservation of spatial context and high-resolution mapping

Spatial omics technology overcomes the limitation of single cell sequencing by preserving the native spatial connections between cells, which enables researchers to examine cell interactions within their natural tissue environment [62]. For instance, Bleckwehl T et al. employed spatial transcriptomics to generate a high-resolution map of human atherosclerotic plaques, elucidating the cellular composition and spatial distribution within atherosclerotic plaques. Their findings revealed that vascular smooth muscle cells migrate toward the lumen and undergo phenotypic transformation into fibro-muscular cells, while lymphocytes are recruited to the lesion site via endothelial cells [63]. Additionally, other investigators utilized a spatial transcriptomic clock to construct a spatial transcriptomic atlas of gene activity throughout the murine brain's lifespan. This approach not only retains spatial relationships between cells but also reveals the contrasting influences of T cells and neural stem cells on neighboring cells [64]. High-resolution mapping techniques, such as IMC and imaging ST, help detect nuanced variations in gene expression and protein concentrations across adjacent cells [46,65]. Collectively, spatial omics techniques are crucial for understanding cell-cell interactions and their impact on aging. By maintaining the spatial orientation of cells in tissues, these methods provide detailed insights into cellular behaviors and interdependencies. This, in turn, opens up new ways to study the complex cellular processes that drive tissue homeostasis and senescence.

#### 3.4.2. Analysis of dynamic interactions

Spatial omics provides insights into the dynamics of cellular communication and signaling pathways, elucidating how cells respond to their microenvironment and influence one another (Fig. 2C) [18]. In myocardial infarction research, Kramann R et al. integrated spatial transcriptomics, single-nucleus transcriptomics, and chromatin accessibility data to generate high-resolution maps of the human heart across distinct time points. This approach enabled the analysis of intercellular interactions during cardiac remodeling [66], revealing significant cell-type-dependent interactions between myofibroblasts and activated macrophages [66]. The recently developed FlowSig model, which integrates single-cell and spatial transcriptomic data to infer directionality within intercellular communication, thereby accurately capturing the spatiotemporal dynamics of cellular signaling [67]. Furthermore, Squidpy is a sophisticated Python-based tool specifically designed for the analysis and visualization of spatial molecular data [68]. It can efficiently store high-resolution images and calculate spatial distances between adjacent points within spatial omics datasets, thereby facilitating the investigation of the spatial distribution of molecules within tissue microenvironments. Collectively, these examples illustrate that spatial omics technologies can help us better understand how cells signal each other during aging and respond to their surroundings.

#### 3.4.3 Integration of multi-omics data

Integrating data from multiple omics dimensions, including genomics, transcriptomics, and proteomics, significantly enhances the comprehensive elucidation of cellular mechanisms and spatial dynamics (Fig. 2B) [62]. For instance, the development of an aging clock, utilizing multi-omics data derived from blood samples, enables the detection of differences in initial aging patterns across various organs or systems [69]. Additionally, the construction of a spatiotemporal atlas of multi-organ aging in mice has enabled the identification of aging core regions and revealed that the accumulation of immunoglobulins is a pivotal hallmark of the aging process. Their study elucidated the intricate network of gene expression, protein distribution, and intercellular interactions in senescent cells [70]. Furthermore, the Aging Atlas database integrates a wide range of aging-related data, including bulk transcriptomics, single-cell transcriptomics, epigenomics, proteomics, and pharmacogenomics, thereby facilitating the convergence and analysis of gene expression regulation trends under diverse aging conditions [71]. This integrative multi-omics approach provides crucial support for both fundamental research and translational applications in the field of aging biology. Recently, Rong Fan at Yale University has made significant contributions to the field of spatial multi-omics, pioneering innovative methodologies for the comprehensive analysis of cellular function and tissue microenvironments. Initially, they introduced the DBiT-seq technique, which enables in situ analysis of spatial transcriptomics and proteomics [72], thereby elucidating the intricate interplay between cellular functions and tissue microenvironments. Building on this foundation, the team further developed two spatial epigenetic (STE) techniques: Spatial-ATAC-RNA-seq and Spatial-CUT&Tag-RNA-seq [73]. These innovations facilitate high-resolution dissection of gene expression regulation mechanisms and enhance our understanding of the complex epigenetic landscape in tissues. Most recently, the team has unveiled the Spatial-CITE-seq technology, which allows for the simultaneous profiling of up to 200 to 300 proteins alongside transcriptomic information [74]. This breakthrough significantly elevated the throughput and precision of multi-omics analysis, positioning it as a powerful tool for systems biology. Given these advancements, we posit that the development of a spatially resolved epigenome–transcriptome co-sequencing technique would represent a transformative milestone in the study of complex tissue biology. This technology, which integrates spatial epigenetics and transcriptomic data, holds the potential for broad applications in key areas of biomedical research, including aging studies. Furthermore, spatial omics technologies are particularly efficacious in investigating complex biological phenomena with spatial heterogeneity, such as tissue development, aging, immune responses, and cancer progression [62,75].

In summary, spatial omics technologies are transforming our understanding of cellular biology by generating detailed, high-resolution maps of molecular activities within tissues, thereby advancing the study of intricate biological systems within their native spatial contexts.

## 4. Spatial Omics in Cardiovascular Aging

Spatial omics elucidates the unique cellular and regional characteristics of cardiac and vascular aging by examining the spatial heterogeneity within cardiovascular tissues. For instance, these technologies enable the mapping of gene and protein expression patterns across distinct cardiac compartments throughout various developmental stages, including the left ventricle, right ventricle, and atrial regions [76,77]. This capability not only facilitates the understanding of changes in gene expression and cellular distribution during cardiac aging but also provides a comprehensive view of how aging affects diverse cell types, including cardiomyocytes, fibroblasts, endothelial cells, and vascular smooth muscle cells.

### 4.1 Spatial context of cardiac cellular senescence

Cardiac aging, influenced by factors such as metabolic stress, oxidative stress, and DNA damage, is characterized by cell cycle arrest, metabolic dysfunction, and the secretion of factors associated with the SASP (Fig. 2C). These changes can substantially impact the surrounding cellular environment, and potentially initiate cardiovascular diseases [9]. Recently, the widespread adoption of single-cell analytical techniques, such as single-cell proteomics, RNA sequencing, and assay for transposase-accessible chromatin (ATAC) sequencing, has revolutionized our understanding of cellular heterogeneity, developmental trajectories and plasticity of cardiovascular pathologies. These techniques elucidate genomic, proteomic, transcriptomic, and metabolomic information at single-cell resolution [15]. For instance, single-cell RNA sequencing (scRNA-seq) and multiplexed error-robust FISH have significantly propelled cardiac aging research by revealing the complex changes within the heart over time [78]. These cutting-edge technologies have revealed previously uncharacterized cardiac cell populations and identified the signaling pathways that coordinate interactions between different cardiac cell types, particularly the plexin-semaphorin pathway involved in ventricular wall morphogenesis [78]. While single-cell technologies inherently lack spatial context, spatial omics techniques preserve the spatial localization of cells in situ [79]. For example, Kanemaru et al. used spatially resolved multi-omics methodologies to investigate distinct cellular microenvironments, or "cell nests," within the heart [80]. They characterized these cellular populations through both scRNA-seq and snRNA-seq and recaptured structural data typically lost through ST [80]. Thus, the integration of single-cell and spatial omics enables the localization of individual cells or specific cellular subpopulations exhibiting particular characteristics within their respective tissue niches. This enhances our understanding of cellular communication within the aging microenvironment. The construction of the most detailed and comprehensive human cardiac cell atlas to date serves as a prime example [78,80]. The researchers employed single-nucleus RNA sequencing (snRNA-seq), ATAC sequencing, and ST to unravel the diverse cellular types and their functional interactions in various states within eight regions of the human heart. Furthermore, they have developed a drug-target prediction tool (Drug2Cell) based on these findings [80], which aims to identify potential therapeutic targets based on the cellular and molecular insights derived from this integrated omics approach.

The Human Cell Atlas (HCA) and the Human Bio Molecular Atlas Program (HuBMAP) exemplify the synergistic application of single-cell and spatial omics to construct comprehensive human cell atlases [81-83]. These mappings provide detailed information on spatial distribution, functional roles, and properties of various cell types in different tissues. The HCA primarily leverages scRNA-seq to delineate gene expression profiles for cell classification, complemented by spatial transcriptomics to map the precise locations of cells within tissues. In contrast, HuBMAP focuses on spatial proteomics and multimodal imaging technologies to elucidate the subcellular localization of proteins and the spatial distribution of cells within tissues. Beyond the aforementioned advancements, Chaffin et al. compared heart samples from patients diagnosed with dilated cardiomyopathy and hypertrophic cardiomyopathy with those from healthy individuals, constructing a comprehensive single-cell atlas of human heart failure employing scRNA-seq. This work has provided a vital reference for identifying heart failure biomarkers and optimizing therapeutic efficacy [84]. The integration of scRNA-seq and bulk RNA sequencing databases has elucidated the gene expression landscape during heart failure [85]. Building upon these advancements, recent studies have leveraged the integration of ST to comprehensively characterize the temporal and spatial variations in gene expression at the cardiac organ level during human morphogenesis resolution [76]. These pioneering studies produced the first organ-wide human developmental transcriptional map, enabling the visualization and analysis of spatiotemporal gene expression patterns in heart development through two- and three-dimensional models [76]. The findings underscore the critical importance of integrating spatial and temporal information with single-cell gene expression datasets to discern key distinctions between cell types and provide an in-depth analysis of developing cardiac tissues [76]. Moreover, certain researchers have employed this same integrative approach to spatially map the trajectory of FAP/POSTN fibroblasts, and revealed that this trajectory is regulated by IL-1β, derived from macrophages, thereby influencing cardiac fibrosis [86]. Moreover, certain researchers used a synergistic approach, integrating ST and STP, to explain pivotal protein influence on cardiac aging, such as sirtuin 2 (SIRT2), which deacetylates signal transducer and activator of transcription 3 (STAT3) to suppress the CDKN2B gene expression [87]. Furthermore, a research consortium harnessed STP, in conjunction with laser capture microdissection and mass spectrometry, to analyze the myocardium and microvasculature across diverse cardiac anatomical regions. Accordingly, they established a comprehensive protein atlas and identified C-C motif chemokine ligand 17 (CCL17) as a novel therapeutic target against cardiovascular aging (Fig. 2E) [88]. Additionally, recent investigations have examined age- and sex-associated transcriptional activity and chromatin accessibility within cardiomyocytes through the analysis of extensive single-cell ATAC-Seq and RNA-Seq datasets [89]. Recent advances in research methodologies have enabled the integration of ST with single-cell ATAC-Seq and RNA-Seq, exemplified by the development of Spatial ATAC-RNA-seq technology. This innovative approach permits the concurrent analysis of chromatin accessibility and gene expression within the same tissue section, thereby providing a robust tool for elucidating cell spatial distribution and gene regulation [73]. These findings significantly enhanced our understanding of how cell type-specific regulatory mechanisms are modified during cardiac aging. Additionally, the newly developed ImAge technology facilitates the capture of the spatial organization of chromatin and epigenetic modifications at the single-cell level, offering valuable insights into chromatin accessibility in senescent cardiac cells and contributing to our understanding of the epigenetic regulation of cellular senescence [90].

In conclusion, spatial omics can reveal the spatiotemporal heterogeneity of age-related alterations, identify regions of senescent cell accumulation, and elucidate how these cells induce tissue dysfunction through their secretion of pro-inflammatory factors [7]. These regions may increase susceptibility to fibrosis and ultimately lead to diminished cardiac function [7]. Furthermore, the reparative and regeneration capacity of aging cardiac tissue is intrinsically associated with its stem cell niches, which often decline or become dysfunctional with advancing age [91,92]. Spatial omics have the potential to elucidate how aging impacts cellular niches and their regenerative potential in the heart and to analyze the distribution of aging-related molecular markers, including inflammatory cytokines, oxidative stress markers, and cell cycle regulators. This comprehensive analysis identifies specific regions of the heart that are disproportionately affected by aging, thereby providing insights into the underlying molecular mechanisms.

### 4.2 Spatial context of vascular cellular senescence

The cellular composition and molecular characteristics of aged human vasculature remain unclear because blood vessel wall cells from various sources are highly heterogeneous [93]. This lack of clarity hinders a comprehensive understanding of the mechanisms underlying vascular aging and limits intervention developments for cardiovascular aging and associated diseases. However, ST has made substantial progress by visualizing and quantifying gene expression within tissues while preserving the original spatial positional information of cells [94], particularly in elucidating the spatial heterogeneity of different cell types and molecular markers. For example, single-cell transcriptome sequencing and spatial cell sorting have been used to study the gene expression profiles and molecular characteristics of vascular cell types in arteries, including endothelial cells, smooth muscle cells, and fibroblasts [93]. These studies identified novel molecular markers distinguishing arterial cell types and revealed that downregulation of the longevity gene *FOXO3A* serves as a driving force for vascular aging [95], with its suppression prominently accelerating this process (Fig. 2D) [95]. Another study employing scRNA-seq techniques demonstrated pronounced heterogeneity in aging vascular endothelial cells, with aging-related transcriptional signatures intersecting with those observed in various vascular pathologies [96]. Furthermore, inflammation is a significant molecular mechanism in aging, and studies have used single-cell transcriptomics and ST to reveal the IL-1β-mediated phenotypic transition of vascular smooth muscle cells during vascular and cardiovascular inflammation, offering a novel perspective for understanding relevant pathological processes [97]. In a recent investigation, researchers explained the pathways associated with proximal vulnerable regions within human atherosclerotic plaques and identified the pivotal gene matrix metalloproteinase 9 (MMP9) via ST, proposing novel therapeutic targets to mitigate plaque rupture [98]. MMP9, a protease within the SASP, is crucial for the extracellular matrix (ECM) remodeling throughout cellular senescence [99,100]. Additionally, other studies have identified differential expression patterns of long non-coding RNAs and mRNAs potentially implicated in aortic aging through high-throughput sequencing methodologies [101]. Furthermore, some researchers have delineated the spatially resolved proteomic landscape of liver endothelium by integrating spatial cell sorting with transcriptomics and quantitative proteomics/phosphoproteomics. This provides intricate mechanistic insights into compartmentalized vascular signaling pathways [102]. Considering the implications of this research, we posit that spatial omics technology may also offer a novel framework, to investigate the compartmentalized signaling mechanisms underlying cardiac endothelial vascular aging. Collectively, these studies emphasize the significant potential of ST technology, in elucidating the molecular and cellular heterogeneity associated with vascular aging, hence providing new directions for understanding its molecular mechanisms and developing preventive and therapeutic strategies.

Recently, the Aging Biomarker Consortium released an authoritative consensus on indicators for vascular aging, proposing a systematic framework that categorizes biomarkers into three dimensions, including functional, structural, and humoral markers [103,104]. The consensus highlights the most clinically pertinent indicators of vascular aging. Spatial omics technologies were not explicitly mentioned within the consensus; however, the multi-dimensional biomarker concept presented in the framework aligns with the type of information provided by spatial omics technologies. Furthermore, this consensus provides groundwork for assessing the extent of vascular aging, with the aim of evaluating individual vascular aging progression (current age), rate of vascular aging (speed of aging), predicting the risk of vascular aging-related diseases (proximity to disease), and other clinical inquiries. Hence, the ultimate objective is to enhance vascular health among older adults in China and globally. Spatial omics technology is anticipated to significantly influence the exploration and investigation of biomarkers relating to vascular aging, with the continuous advancement of technology and broadening of its applications.

**Figure 3.**
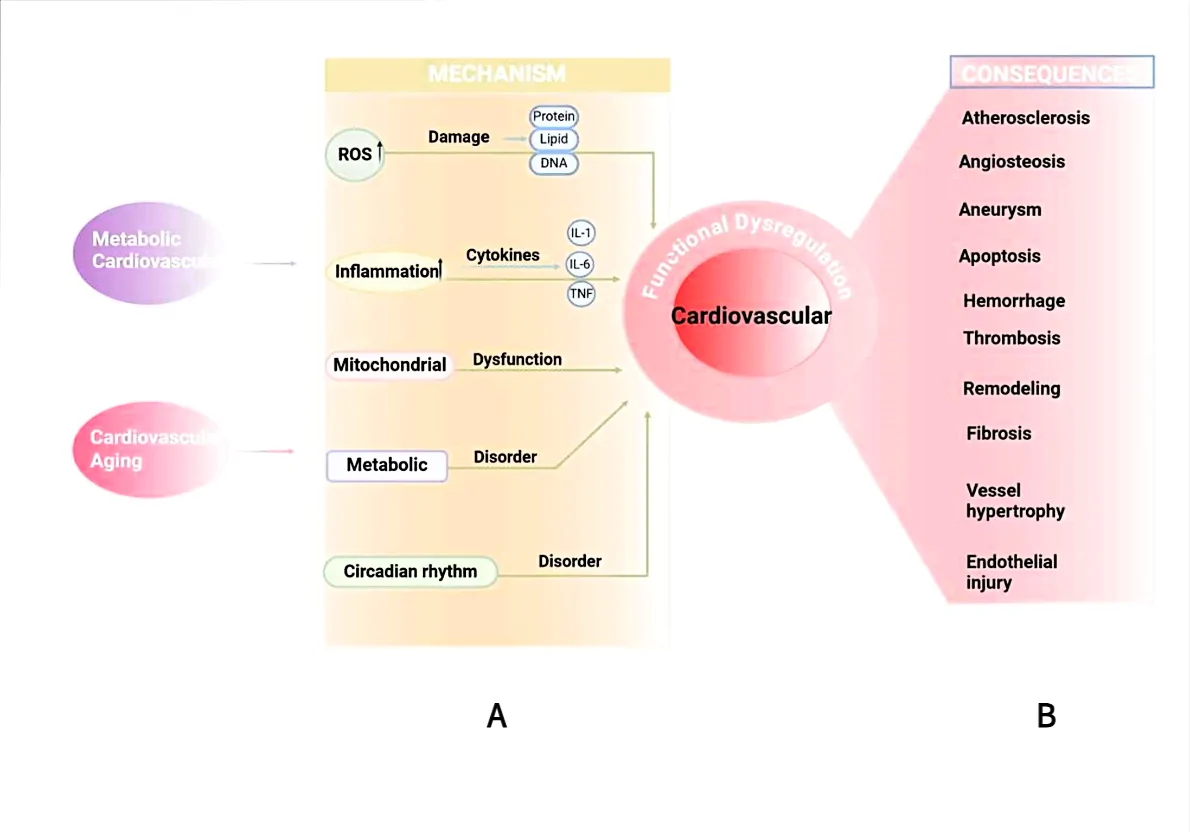
**The relationship between metabolic cardiovascular and cardiovascular aging. (A)** Metabolic cardiovascular disease and cardiovascular aging are interconnected through shared molecular pathways, characterized by heightened oxidative stress, persistent inflammation, metabolic dysregulations, mitochondrial dysfunction, and circadian rhythm disturbances. **(B)** The mechanisms at play within the cardiovascular system culminate in pathophysiological outcomes, including atherosclerosis, aneurysm, angiostenosis, vessel hypertrophy, endothelial injury, thrombosis, hemorrhage, apoptosis, vascular fibrosis, and remodeling.

### 4.3 Interplay between metabolic disease and cardiac aging

Metabolic cardiovascular disease and cardiovascular aging are closely intertwined. They are characterized by heightened oxidative stress, persistent inflammation, metabolic dysregulations, mitochondrial dysfunction, and circadian rhythm disturbances (Fig. 3A) [56,105]. Oxidative stress induces the generation of reactive oxygen species, which inflict damage on lipids, proteins, and DNA [9], thereby contributing to the pathophysiological processes, such as inflammation and metabolic dysregulations (Fig. 3B) [6,106,107]. Furthermore, persistent inflammation is a salient feature of cardiovascular aging [6], and often acts in concert with metabolic disturbances to drive the progression of metabolic cardiovascular diseases and cardiovascular aging [105], such as diabetic cardiovascular disease and the progression of atherosclerosis [9,108]. Inflammatory cytokines such as IL-1β, IL-6, and tissue necrosis factor are often involved in cardiovascular aging and metabolic cardiovascular disease [6,109]. Other metabolic disorders, such as insulin resistance and lipid dysregulation, also adversely affect cardiac function and vascular health [7,110,111]. Additionally, mitochondrial dysfunction accelerates cardiovascular aging processes by increasing oxidative stress, inhibiting autophagy and energy metabolism, and severely affecting cardiovascular metabolism, increasing the risk of age-related cardiovascular diseases [6,105]. Furthermore, diurnal rhythm plays an important regulatory role in the physiological processes of the cardiovascular system [112]. Certain clock genes such as *Bmal1* and *Per2* are crucial in cardiovascular protection [113]. Cardiovascular metabolism and aging are both influenced by the circadian rhythms. For example, a slave clock, Krüppel-like factor 15, integrates signals from the core clock (BMAL1) and external metabolic cues—such as diet, exercise, heart disease, and aging—to directly regulate the expression of many metabolic enzymes during their active phases [114]. Moreover, mutations in the *Per2* gene, which are associated with regulating diurnal rhythm, may induce vascular aging via the AKT (Protein Kinase B) signaling pathway [115]. Additionally, disruption of the diurnal rhythm leads to immune cells or stem cell aging [116,117], thereby exerting detrimental effects on cardiovascular aging. Notably, one investigation utilizing advanced metabolomic techniques revealed that the metabolic signatures of human aging encompass multiple pathways, including steroid, amino acid, lipid, and purine metabolism [118]. Researchers can use STM to discern the aberrant accumulation or depletion of metabolites within specific cell types or tissue regions in cardiovascular aging. These metabolites are associated with key metabolic pathways and new biomarkers during circadian rhythm disruption. This spatially resolved metabolic profiling facilitates the identification of localized metabolic impacts of circadian rhythm disturbances, as well as cellular responses to metabolic changes. Thus, it provides deeper insights into the intricate interplay between these two phenomena. Similarly, another study revealed that metabolomics, through its ability to measure a diverse range of metabolites with distinct chemical characteristics, holds the potential to capture both the biochemical changes induced by pathological processes and the natural progression of aging [119]. Moreover, metabolomics, when integrated with non-invasive cardiovascular imaging techniques, can facilitate a comprehensive understanding of the detrimental consequences of cardiovascular aging [119]. Spatial omics serves as a unified framework that permits the examination of the interplay between metabolic cardiovascular diseases and cardiovascular aging. By combining spatial omics with other modalities, such as proteomics and metabolomics, researchers can obtain spatial distribution data for proteins and metabolites. They can also identify novel biomarkers and therapeutic targets and elucidate the metabolic changes and cellular senescence patterns across different heart regions. This multi-dimensional approach enables a more holistic understanding of disease mechanisms, thereby advancing the development of targeted therapies and personalized medicine.

## 5. Clinical Implications and Future Perspectives

### 5.1 Therapeutic opportunities

Recently, scholars utilizing single-cell whole-genome sequencing have discovered that somatic single nucleotide variations gradually accumulate in cardiac myocytes with aging [120]. Another longitudinal multi-omics study, including transcriptomics, proteomics, metabolomics, cytokine profiling, and microbiome analysis, has revealed significant nonlinear alterations throughout the aging process, particularly at the pivotal junctures of 40 and 60 years of age [42]. These changes involve pathways related to cardiovascular health, lipid metabolism, immune function, and oxidative stress response [42]. These observations underscore that aging extends beyond mere temporal progression, encompassing dynamic spatial alterations within cells and tissues. The integration of single-cell omics and spatial omics technologies constitutes a formidable toolkit for investigating cardiovascular aging. This amalgamation precisely delineates the distribution of senescent cells within cardiovascular tissues, their interactions with neighboring cells, and the consequent impact on overall functionality.

**Figure 4.**
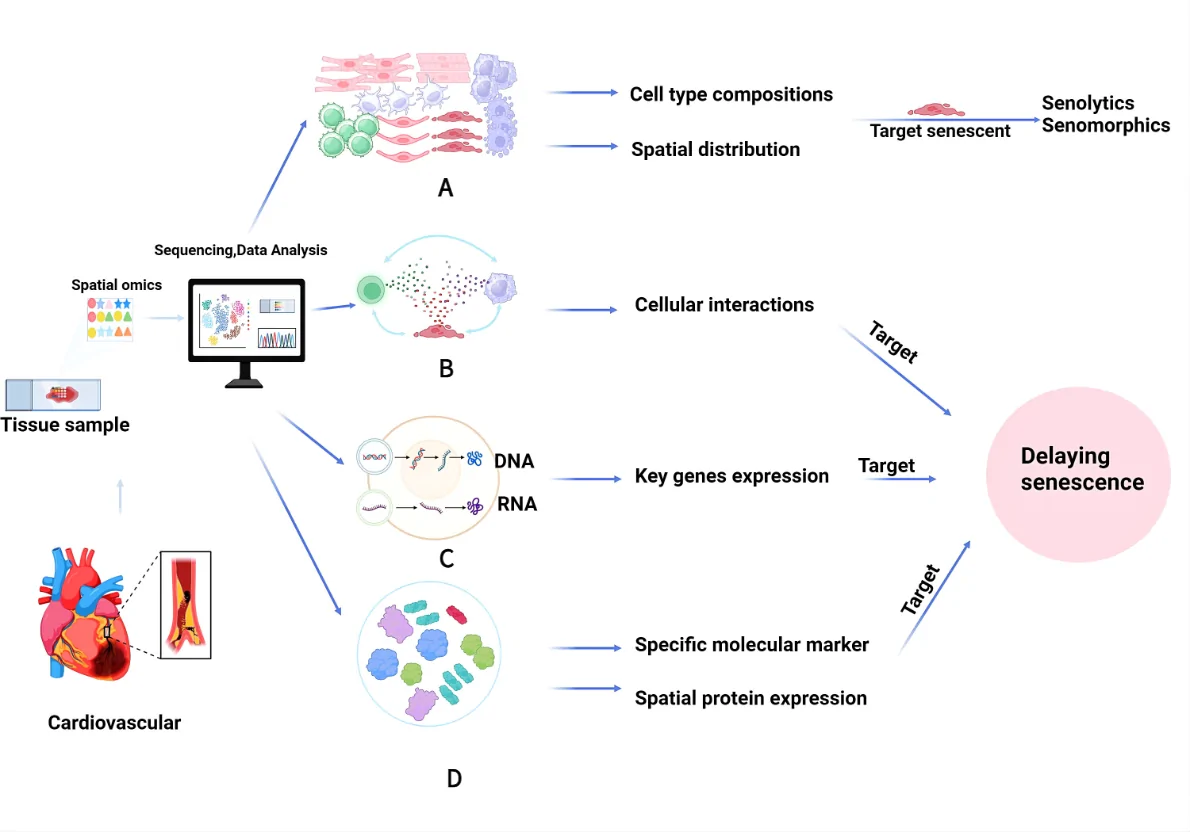
**Spatial omics not only help identify the compositions, spatial distribution, and cellular interactions of different cell types within the cardiovascular system but also aid in examining key genes, specific molecular markers, and spatial protein expression within these regions: (A)** By identifying regions of accumulated senescent cells to formulate targeted senolytics and senomorphics. **(B)** By studying cellular interactions of different cell types to develop targeted interventions in communication networks. (**C)** By determining key genes to design targeted gene therapy. **(D)** By examining specific protein molecular markers, we can target related molecular pathways, thus formulating targeted therapeutic approaches.

For instance, MERFISH and Xenium technologies are emerging as powerful tools to elucidate the molecular mechanisms of cardiac senescence. Multiplexed Error-Robust Fluorescence In Situ Hybridization (MERFISH), with its high-resolution imaging and multiplexed gene detection [121], enables the identification of upregulated and downregulated genes in aged cardiac tissues. It delineates regions of senescent cell aggregation and elucidates their transcriptional profiles. Moreover, MERFISH can also be integrated with scRNA-seq to provide a more comprehensive understanding of cardiovascular aging. By identifying local clusters of senescent cells associated with tissue dysfunction, these technologies offer insights for developing targeted therapeutic strategies, such as lysosomal therapies aimed at selectively eliminating senescent cells or modulating their secretory phenotype (Fig. 4A) [9,122]. Furthermore, investigators have successfully identified innovative therapeutic targets associated with cardiovascular aging, employing single-cell sequencing and ST, such as CCL17 [88]. CCL17 influences vascular aging by modulating T cell activity, and its inhibition can potentially mitigate vascular fibrosis and other age-related lesions, thereby offering a promising new avenue for the therapeutic management of cardiovascular aging [88]. Xenium, based on a padlock probe and rolling-circle amplification, offers high-sensitivity detection of gene expression changes. It can discern even subtle variations, such as splice variants and SNPs [123]. This technology is adept at monitoring expression changes within aged hearts. Its non-destructive workflow allows integration with histopathological data, enabling detailed analysis of the spatial distribution of aging-associated genes.

Furthermore, this approach enables researchers to observe the interactions between senescent cells and the extracellular matrix (ECM) at the tissue level [124], as the accumulation of senescent cells can alter ECM composition and trigger the SASP [99]. This analysis elucidates how senescent cells influence neighboring cells through the secretion of pro-inflammatory cytokines and chemokines, thereby contributing to cardiovascular aging (Fig. 4B).

STE technologies, which include Spatial ATAC–RNA-seq or Spatial CUT&Tag–RNA-seq, can reveal changes in chromatin states and histone modifications within aged cardiovascular tissues (e.g., the distribution of H3K27me3 and H3K27ac) [73]. These technologies offer insights into the role of epigenetic regulators in aging, such as MLF1-mediated chromatin remodeling in cardiomyocyte senescence [125]. Integrating epigenetic modifications with gene expression data provides a comprehensive understanding of how gene expression is regulated epigenetically and uncovers cell type-specific and region-specific gene regulation. Additionally, STP enables the study of protein distribution and expression changes in aged cardiovascular tissues, such as identifying aging-related proteins and potential biomarkers (e.g., SIRT2). For instance, researchers have developed a novel technology termed DISCO-MS, which is capable of precisely identifying “diseased” cells at the early stages of pathology and obtaining proteomic data therefrom. This advancement holds the potential to reverse the functional impairments associated with cardiovascular aging [126]. Reversal of cardiovascular aging may be achieved through targeted gene therapy directed at these key proteins (Fig. 4C-D). STM, on the other hand, can identify changes in metabolites within aged cardiovascular tissues (e.g., the accumulation of lipid metabolites) and elucidate the relationship between metabolites and cellular senescence.

In summary, spatial omics technologies transcend mere molecular analysis by precisely localizing cells and tissue architecture and integrating complex molecular information with spatial coordinates. These technologies provide a comprehensive molecular mapping of the cardiovascular tissue microenvironment and allow researchers to visualize dynamic changes in genes, epigenetic modifications, proteins, and metabolites during aging, thereby revolutionizing the identification of novel therapeutic targets for cardiovascular aging. This advancement provides a novel dimension to the study of cardiovascular aging, empowering researchers to elucidate the aging process from a spatial perspective.

### 5.2 Challenges and the road ahead

The translation of spatial omics technologies into clinical applications for cardiovascular aging research encounters multifaceted challenges, including technical complexity, high cost, data integration, standardization, and data acquisition [22,60]. For instance, difficulties in image registration and the inability of spatial barcodes to precisely correspond to individual cells impede the realization of gene expression matrices [16,127]. A novel approach known as STIE is proposed to address the gap between junction resolution and single-cell level analysis, providing spatial localization information of gene expression by integrating spatial transcriptome data with corresponding histological images [128]. This approach mitigates the limitations of spatial barcoding techniques and reveals the heterogeneity of cardiovascular aging. Furthermore, the integration of spatial omics data with existing clinical datasets and diagnostic tools presents another significant challenge, highlighting the critical need for the development of algorithms and platforms capable of seamlessly merging these datasets. Additionally, the absence of unified standards in sample preparation, data acquisition, and analysis processes undermines the comparability across studies. Therefore, establishing standardized protocols for sample preparation, data collection, and analysis is important to ensure the reliability and reproducibility of spatial omics within clinical research and practice [127,129]. Future opportunities lie in technological innovation, multi-omics integration, biomarker identification, precision medicine, therapeutic target discovery, and clinical trials. Advances in spatial omics technologies are anticipated to enhance resolution, sensitivity, and throughput, thereby achieving a more precise subcellular level and rendering them more applicable in clinical settings [11,22]. The integration of spatial omics with diverse omics data—such as genomics, proteomics, and metabolomics, necessitates high-performance computing and novel algorithms. However, the existing tools are still immature. Further refinement of algorithmic tools would facilitate the development of more precise diagnostic tools and personalized therapeutic strategies.

Moreover, spatial omics can potentially uncover novel biomarkers pertinent to cardiovascular aging. These biomarkers are crucial for early detection, risk stratification, and monitoring of therapeutic responses. These technologies may also contribute significantly to advancing precision medicine by providing intricate molecular mapping. This mapping reflects individual variability in cardiovascular aging processes and guides the formulation of targeted therapies. By elucidating the spatial distribution of molecular alterations associated with cardiovascular aging, spatial omics can help identify specific pathological mechanisms. The integration of artificial intelligence and machine learning algorithms holds the potential to facilitate a more profound analysis of spatial omics data. Such approaches may elucidate concealed pathways associated with aging and identify novel therapeutic targets. Incorporating spatial omics into clinical trial frameworks may facilitate the evaluation of novel treatments for cardiovascular aging. It can also help identify patient cohorts most likely to derive benefit from such interventions. While the clinical translation of spatial omics in cardiovascular aging research presents certain challenges, its prospective advantages are substantial. Through continuous technological refinement, standardization efforts, and collaborative initiatives, spatial omics is poised to become a valuable asset in our understanding and management of cardiovascular aging.

## 6. Conclusion

Spatial omics technology has revolutionized the study of cardiovascular aging by enabling the precise mapping of gene expression, protein distribution, and metabolite levels within different regions of cardiovascular tissue. This advancement elucidates the spatial dimension of age-related molecular alterations and cellular heterogeneity. Additionally, these technologies facilitate the identification of novel biomarkers pertaining to cardiovascular aging, thereby supporting early diagnosis, risk assessment, and tracking the progression of age-related changes. Moreover, it pinpoints novel therapeutic targets and propels the development of personalized medicine. The integration of spatial omics into cardiovascular aging research represents a significant stride in the early detection, prevention, and treatment of age-related cardiovascular diseases.

## Data Availability

The authors declare that all data presented in this study will be presented upon request from the corresponding author.
